# Population genetics and genetic variation of *Pomacea canaliculata* (Gastropoda: Ampullariidae) in China revealed by sequence analyses of three mitochondrial genes

**DOI:** 10.1002/ece3.10836

**Published:** 2024-01-17

**Authors:** Xi‐Long Yi, Jing Liu, Mei‐Ling Cao, Jun Xiong, Yuan‐Ping Deng, Hui‐Mei Wang, Ping‐Ping Ma, Guo‐Hua Liu, Hua Yang

**Affiliations:** ^1^ Research Center for Parasites & Vectors, College of Veterinary Medicine Hunan Agricultural University Changsha Hunan Province China; ^2^ College of Bioscience and Biotechnology Hunan Agricultural University Changsha Hunan Province China

**Keywords:** genetic diversity, mitochondrial DNA, phylogenetic analysis, *Pomacea canaliculata*

## Abstract

The Golden apple snail, *Pomacea canaliculata*, is one of the world's 100 worst invasive alien species that is best known for its damage to wetland agriculture. It also acts as an intermediate host of some zoonotic parasites such as *Angiostrongylus cantonensis*, posing threats to human public health and safety. Despite is being an important agricultural pest, the genetic information and population expansion history of this snail remains poorly understood in China. In this study, we analyzed the genetic variation and population genetics of *P. canaliculata* populations in seven regions of China based on molecular markers of three mitochondrial (mt) genes. A total of 15 haplotypes were recognized based on single mt *cox1*, *nad1*, and *nad4*, and eight haplotypes were identified using the concatenated genes. High haplotype diversity, moderate nucleotide diversity, low gene flow, and high rates of gene differentiation among the seven *P. canaliculata* populations were detected. Shanghai and Yunnan populations showed higher genetic flow and very low genetic differentiation. The results of Tajima's *D*, Fu's *F*_s_, and mismatch distribution showed that *P. canaliculata* did not experience population expansion in China. Genetic distance based on haplotypes suggested that *nad1* gene was more conserved than *cox1* gene within *P. canaliculata*. The phylogenetic analyses showed there may be two geographical lineages in the Chinese mainland. The present study may provide a new genetic marker to analyze *P. canaliculata*, and results support more evidence for studying the genetic distribution of *P. canaliculata* in China and contribute to a deeper understanding of its population genetics and evolutionary biology.

## Graphical abstract

The Golden apple snail, *Pomacea canaliculata*, is one of the world's 100 worst invasive alien species that is best known for its damage to wetland agriculture. We analyzed the genetic variation and population genetics of *P. canaliculata* populations in seven regions of China based on molecular markers of three mitochondrial (mt) genes.

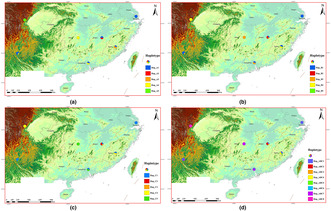

## 1 INTRODUCTION

*Pomacea* (Gastropoda: Ampullariidae) is an invasive freshwater snail genus distributed mainly in tropical and subtropical regions where it is warm and humid (Hayes et al., 2008). It is native to South America, and was introduced to southern and eastern Asia as aquarium pets or human food after the 1980s (Hayes et al., 2008; Rawlings et al., 2007). *Pomacea canaliculata* (Lamarck 1822) and *Pomacea maculata* (Perry 1810), two representatives in the genus *Pomacea*, are the most invasive *Pomacea* species, particularly *P. canaliculata*, which was listed by the Invasive Species Specialist Group (ISSG) as one of the 100 most invasive alien species worldwide (Lowe et al., 2000). *P. canaliculata* has a flexible diet, not only eating the leaves and stems of seedlings of many important economic aquatic crops (Carlsson et al., 2004; Cowie, 2002) but also consuming other aquatic animals (Carlsson et al., 2004; Guo & Zhang, 2014; Kwong et al., 2009). It also has high fecundity, adaptability, and fast growth, which enables it to survive extreme environmental conditions such as dryness, cold, high salinity, and even heavy metal pollution (Mao et al., 2018). Due to a lack of natural predators and the mild competitiveness of local snails, *P. canaliculata* becomes the dominant population and serious agricultural pest, which seriously endangers local biodiversity and causes huge economic losses (Lv et al., 2011; Mao et al., 2018).

*Pomacea canaliculata* was recorded as one of the important invasive alien species under the first national management in China. In southern China, there are natural *P. canaliculata* populations that have been documented in at least 11 provinces (Yang, Liu, He, & Yu, 2018). With accelerating global warming, the southern Chinese population of golden apple snails is expanding and appears to have a tendency for northward expansion (Lv et al., 2011; Yin et al., 2022). In addition, *P. canaliculata* also acts as the intermediate host of many important zoonotic parasites. It is also understood that *P. canaliculata* may facilitate the spread of *Angiostrongylus cantonensis*, the causative agent of angiostrongyliasis, which causes eosinophilic meningitis (Jian et al., 2012). A report in 2011 showed a small outbreak of angiostrongyliasis caused by consuming *P. canaliculata* (Deng et al., 2011). Under laboratory conditions, experimental results suggested apple snails are suitable hosts and vectors for infective third‐stage larvae of *Gnathostoma spinigerum*, the causative agent of Gnathostomiasis in humans, and that L3 larvae can encapsulate in the tissues and organs of snails (Komalamisra et al., 2009).

The species classification of *Pomacea* species is mainly based on morphological features and molecular information. Morphological characteristics are differentiated by egg colors and overall shapes (spire, apex and mouth of shells, body whorl, shell colors, etc.). However, identifying different *Pomacea* species is challenging due to their similar external shapes (Cazzaniga, 2002). Additionally, snails from the same species can be hard to recognize as their shapes change with invaded environments (Hayes et al., 2015). Recently, molecular biology techniques have facilitated the widespread use of DNA sequences for the analysis of phylogenetic and evolutionary relationships among species, particularly mitochondrial (mt) DNA, which allows inference of population structure and variation and evolutionary analysis because of its structural features (Moritz et al., 1987). The severe impacts and diseases of *P. canaliculata* have been frequently documented; however, the local populations and phylogenetic distances of apple snails in China are still unclear, with only a few reports available, mainly based on a single *cox1* gene (Dumidae et al., 2021; Huang et al., 2020; Yang, Liu, He, & Yu, 2018). Considering multiple‐genes may be more accurate for inferring phylogenetic relationships and population histories, our objectives are to investigate the genetic variation, population genetics, and phylogenetic relationships of *P. canaliculata* from different regions using three mt genes (*cox1*, *nad1*, and *nad4*).

## 2 METHODS

### 2.1 Specimen collection and DNA extraction

**FIGURE 1:**
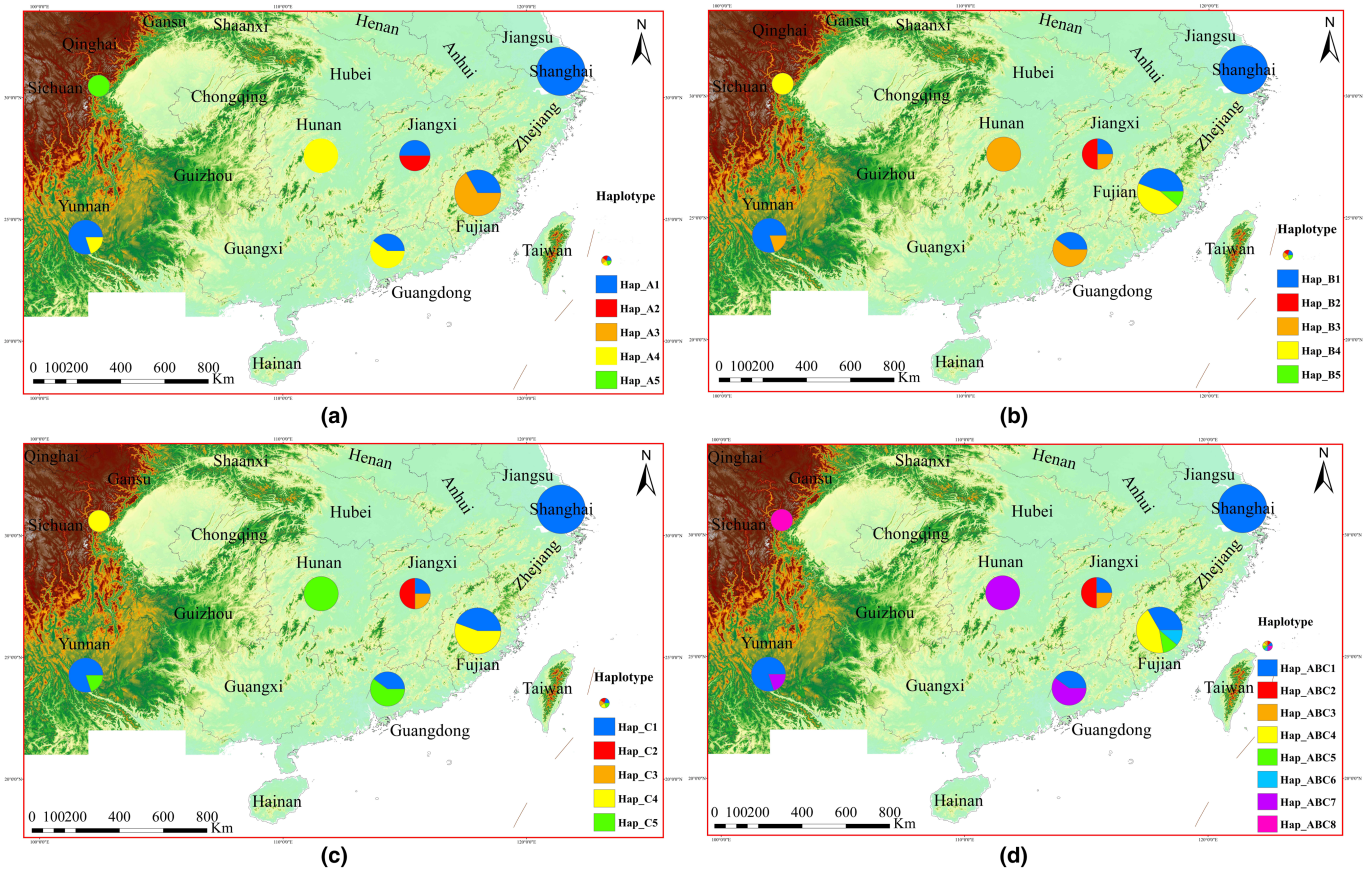
Geographical distribution and frequency of haplotypes of *Pomacea canaliculata* ((a) haplotypes of *cox1*; (b) haplotypes of *nad1*; (c) haplotypes of *nad4*; (d) haplotypes of the concatenated genes) in China. Maps were constructed in ArcGIS 10.8 software (ESRI Inc., Redlands, CA, USA). URL http://www.esri.com/software/arcgis/arcgis‐for‐desktop. The relevant colors in the pie chart indicate haplotype frequencies for each sampling point.

Samples were collected from seven different provinces in southern China (Figure 1). A total of 40 snails were obtained. All samples were then shelled and washed by ultra water, and the soft tissues were stored in 70% ethanol at −40°C until extraction of DNA. Genomic DNA was extracted from 0.5 g of tissue from each sample by standard sodium dodecyl sulfate/proteinase K treatment and phenol/chloroform extraction, followed by column‐purification using the Wizard® SV Genomic DNA Purification System (Promega, USA).

### 2.2 Amplification and sequencing

Using available complete mt sequence of *P. canaliculata* (GenBank Accession No. KY008700) as reference, three pairs of PCR primers were designed (Table 1) to amplify the mt *cox1*, *nad1* and *nad4* genes, respectively. PCR was performed in the C1000 Touch™ Thermal Cycler (BioRad, USA) with 25 μL reactions containing 1 μL of each primer, 9.5 μL ddH_2_O, 12.5 μL Premix Taq™ (Takara, LA Taq™ V2.0 plus dye), and 1 μL of DNA template. The conditions were set as following: an initial denaturation at 94°C for 5 min, then 94°C for 30 s, 50°C (for *cox1*) or 55°C (for *nad1*) or 54°C (for *nad4*) for 30 s, and 72°C for 1 min for 35 cycles, and a final extension at 72°C for 7 min. PCR products were visualized by electrophoresis in 1.5% agarose gel and then sent to Tsingke Biotechnology Co., Ltd (Changsha, Hunan, China) for sequencing for both directions.

**TABLE 1:** Sequences of primers used to amplify a portion of the *cox1*, *nad1*, and *nad4* of *Pomacea canaliculata* in the present study.

Name of primer	Sequence (5′–3′)	Length (bp)
*cox1*F	TCCTGGCTTTGGGATGATTTCTC	589
*cox1*R	TGGATAATCAGAATAACGACGAG	
*nad1*F	GTATCCCACAACCTATTGCTGAC	436
*nad1*R	CAAGTGGCAAACCATACGAAAGC	
*nad4*F	GGCTGAGGATACCAACCAGAACG	338
*nad4*R	CGTAAGATTCCATAACTCCCTAA	

### 2.3 Sequences preparations and phylogenetic analyses

Sequences were aligned using the Clustal W program in MEGA v.5.0 and then manually cut (Thompson et al., 1994). During this procedure, any ambiguous regions within these alignments were filtered with Gblocks 0.91 b (Castresana, 2000). A neighbor‐joining (NJ) phylogenetic tree was plotted using MEGA5 (Tamura et al., 2011). The NJ bootstrap values were estimated using 1000 replicates with Kimura's two‐parameter model of substitution (K2P distance) (Kimura, 1980). Gaps and missing data were eliminated. A maximum likelihood (ML) analysis was run in PhyML 3.0, using a HKY + G + I model with parameters estimated by the program (Guindon et al., 2010). *P. diffusa* (NC_041142), *P. maculata* (MF401379), and *P. occulta* (KR350466) were also included in the present study, with *Euspira pila* (NC_046703) as the outgroup. In addition, MEGA 5 was also used to calculate the pairwise distance based on Kimura 2‐parameter (K2P) model (Kimura, 1980). Phylograms were drawn using FigTree v.1.3.1 (http://tree.bio.ed.ac.uk/software/fgtree/).

### 2.4 Population differentiation

Genetic diversity values, including haplotype numbers, haplotype diversity (*Hd*), nucleotide diversity (*Pi*), polymorphic sites, and mismatch distribution of each gene were calculated using DnaSP v5.0 (Librado & Rozas, 2009). Based on the same program, pairwise index of genetic differentiation (*F*_st_) were also estimated, and the corresponding gene flows (*N*_m_) between populations were indirectly calculated as follows: *N*_m_ = (1 − *F*_st_)/4*F*_st_ (Nei, 1978; Wright, 1949). Haplotype identification, Tajima's *D* (Tajima, 1989), Fu's *Fs* tests (Fu, 1997) were performed on seven different populations using Arlequin v3.0. In addition, genetic differentiation in seven different populations was also estimated using analysis of molecular variance (AMOVA) (Excoffier et al., 2005). DnaSP 5.0 and PopART 1.7 were used to create a statistically parsimonious network to infer haplotype relationships (Leigh & Bryant, 2015).

## 3 RESULTS

### 3.1 Sequence analyses

All sequences for *nad1*, *cox1*, and *nad4* genes of 40 samples were successfully amplified. The average sizes of *cox1*, *nad1*, and *nad4* were 447, 339, and 264 bp, respectively. All three gene sequences showed high similarities with available *P. canaliculata* sequences in GenBank, indicating all samples were from *P. canaliculata* species. The A+T contents of *cox1*, *nad1*, and *nad4* sequences were 68.5%–69.6%, 71.6%–72.5%, and 68.6%–71.3%, respectively, which were significantly higher than the G+C contents. The detailed information such as sample codes, collection region, collection date, and GenBank accession were listed in Table 2.

**TABLE 2:** The information of collected samples.

Sample ID	Geographical origin	Month	GenBank accession number		
			*cox1*	*nad1*	*nad4*
Shanghai1–Shanghai10	Chongming, Shanghai	August	OQ983595–OQ983604	OQ982487–OQ982496	OQ982527–OQ982536
Jiangxi1–Jiangxi4	Nanchang, Jiangxi	August	OQ983605–OQ983608	OQ982497–OQ982500	OQ982537–OQ982540
Fujian1–Fujian9	Jianou, Fujian	July	OQ983609–OQ983617	OQ982501–OQ982509	OQ982541–OQ982549
Yunnan1–Yunnan5	Honghe, Yunnan	August	OQ983618–OQ983622	OQ982510–OQ982514	OQ982550–OQ982554
Sichuan1–Sichuan2	Chengdu, Sichuan	July	OQ983623–OQ983624	OQ982515–OQ982516	OQ982555–OQ982556
Guangdong1–Gunagdong5	Guangzhou, Guangdong	July	OQ983625–OQ983629	OQ982517–OQ982521	OQ982557–OQ982561
Hunan1–Hunan5	Changsha, Hunan	June	OQ983630–OQ983634	OQ982522–OQ982526	OQ982562–OQ982566

### 3.2 Genetic diversity

**FIGURE 2:**
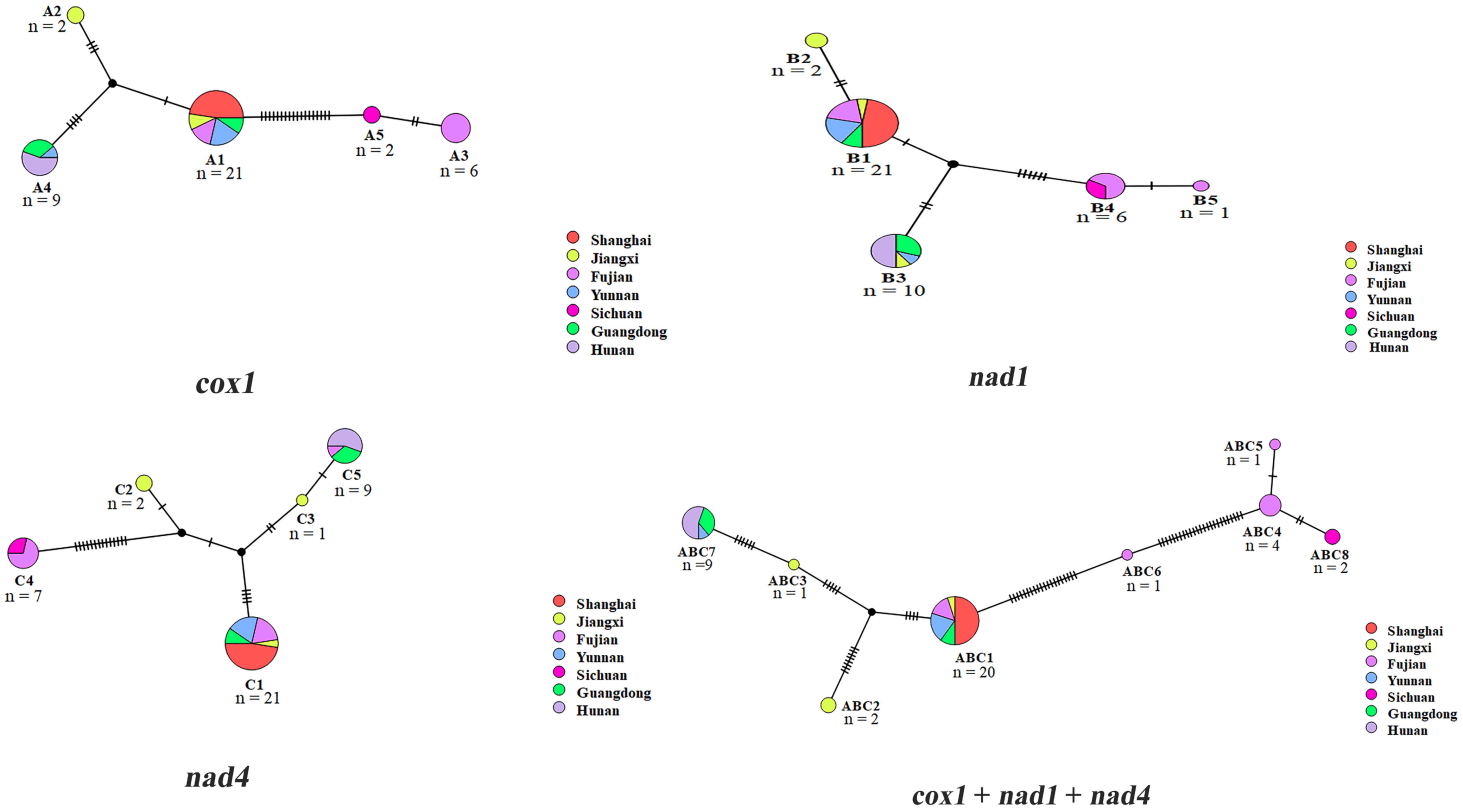
Network maps of *cox1*, *nad1*, *nad4* and the concatenated genes haplotypes of *Pomacea canaliculata*.

The haplotype networks of three partial genes and concatenated genes were displayed in Figure 2. For *cox1*, *nad1*, and *nad4* genes, a total of 15 haplotypes were detected, and eight haplotypes were observed in the concatenated genes (Figures 1 and 2, Table 3). For single gene, haplotype A1 was shared by five populations including Shanghai, Jiangxi, Fujian, Yunnan, and Guangdong, the same with haplotype B1 for *nad1* gene and haplotype C1 for *nad4* gene, suggesting there might be an ancestral haplotype existing among those populations. In addition, haplotypes A4, B3, B4, C4, and C5 were shared by at least two populations. For haplotypes A4 and B3, they were shared by Hunan, Guangdong, and Yunnan populations, while B3 also existed in Jiangxi population. Haplotypes B4 and C4 were common in Sichuan and Fujian populations. C5 haplotype was shared by Hunan, Guangdong, and Fujian. Except for the haplotypes mentioned above, the remaining haplotypes were unique within Jiangxi, Fujian, and Sichuan populations. For the concatenated genes, it comprised of two shared haplotypes (ABC1 and ABC7) and six unique haplotypes. Among those haplotypes, the haplotype ABC1, which was common among Shanghai, Jiangxi, Fujian, Yunnan, and Guangdong, was similar to A1, B1, and C1. As the same as A1, the ABC7 also showed in Hunan, Guangdong, and Yunnan populations. For the *cox1* gene, a total of 27 variable sites were detected, with numbers ranging from 0 to 20. Fujian had the highest number of variables sites, while Shanghai, Sichuan, and Hunan had the lowest. Consistent with the results of *cox1*, the variable sites of *nad1*, *nad4*, and the concatenated genes in Fujian remained dominant (Table 4). Populations as a whole presented high haplotype diversity, with *Hd* values ranging from 0.653 to 0.700, and a moderate nucleotide diversity, with *Pi* values between 0.009 and 0.028 (Table 4). Among the three mt genes, *nad4* was the most varied with the highest nucleotide diversity variation (*Pi* = 0.028), followed by *cox1* (*Pi* = 0.019). Compared with *cox1* and *nad4* genes, *nad1* seemed to be the most stable gene in golden apple snail with a low nucleotide diversity (*Pi* = 0.010). In terms of Shanghai, Sichuan, and Hunan populations, the haplotype diversities and nucleotides diversities were both 0. For the other four populations (Jiangxi, Fujian, Yunnan, and Guangdong), haplotype diversities ranged from 0.040 to 0.833, with the highest *Hd* was observed in Jiangxi population. Nucleotide diversities ranged from 0.004 to 0.222, with the population from Fujian was the highest in both single gene and the concatenated genes.

**TABLE 3:** Haplotype distribution of *Pomacea canaliculata* from China based on *cox1*, *nad1*, *nad4*, and concatenated genes corresponding to the seven sampling locations.

Region	No. of ind (*n*)	Haplotype (*n*)			
		*cox1*	*nad1*	*nad4*	*cox1* + *nad1* + *nad4*
Shanghai	10	A1(10)	B1(10)	C1(10)	ABC1(10)
Jiangxi	4	A1(2), A2(2)	B1(1), B2(2), B3(1)	C1(1), C2(2), C3(1)	ABC1(1), ABC2(2), ABC3(1)
Fujian	9	A1(3), A3(6)	B1(4), B4(4), B5(1)	C1(4), C4(5)	ABC1(3), ABC4(4), ABC5(1), ABC6(1)
Yunnan	5	A1(4), A4(1)	B1(4), B3(1)	C1(4), C5(1)	ABC1(4), ABC7(1)
Sichuan	2	A5(2)	B4(2)	C4(2)	ABC8(2)
Guangdong	5	A1(2), A4(3)	B1(2), B3(3)	C1(2), C5(3)	ABC1(2), ABC7(3)
Hunan	5	A4(5)	B3(5)	C5(5)	ABC7(5)

*Note*: The number in parentheses refers to the relative frequency of the given haplotype.

**TABLE 4:** Variable sites, haplotype, nucleotide diversity, haplotype diversity, Tajimas *D* and Fu's *Fs* based on *cox1*, *nad1*, *nad4*, and concatenated genes of *Pomacea canaliculata* from China.

Gene	Regions	*S*	*H*	*Hd*	*Pi*	Tajima's *D*	Fu's *Fs*
*cox1*	Shanghai	0	1	0	0	–	–
	Jiangxi	4	2	0.667	0.001	2.080	2.718
	Fujian	20	2	0.500	0.222	1.765*	10.686**
	Yunnan	5	2	0.400	0.005	−1.123	2.639
	Sichuan	0	1	0	0	–	–
	Guangdong	5	2	0.600	0.007	1.685*	3.526
	Hunan	0	1	0	0	–	–
	Total	27	5	0.663	0.019	1.091	11.451
*nad1*	Shanghai	0	1	0	0	–	–
	Jiangxi	5	3	0.833	0.008	0.371	0.646
	Fujian	8	3	0.667	0.012	1.800*	3.795*
	Yunnan	3	2	0.400	0.004	−1.048	1.687
	Sichuan	0	1	0	0	–	–
	Guangdong	3	2	0.600	0.005	1.572*	2.428
	Hunan	0	1	0	0	–	–
	Total	12	5	0.653	0.010	0.514	3.930
*nad4*	Shanghai	0	1	0	0	–	–
	Jiangxi	8	3	0.833	0.016	−0.068	1.285
	Fujian	18	2	0.556	0.037	2.491***	10.686***
	Yunnan	7	2	0.400	0.010	−1.161	3.366
	Sichuan	0	1	0	0	–	–
	Guangdong	7	2	0.600	0.016	1.742*	4.360*
	Hunan	0	1	0	0	–	–
	Total	22	5	0.656	0.028	1.297	10.250
*cox1* + *nad1* + *nad4*	Shanghai	0	1	0	0	–	–
	Jiangxi	17	3	0.833	0.009	0.617	2.596
	Fujian	46	4	0.750	0.024	2.159*	8.721***
	Yunnan	15	2	0.400	0.006	−1.218	5.342*
	Sichuan	0	1	0	0	–	–
	Guangdong	15	2	0.600	0.009	1.827**	6.570**
	Hunan	0	1	0	0	–	–
	Total	61	8	0.700	0.018	1.133	15.969

Abbreviations: *H*, number of haplotypes; *Hd*, haplotype diversity; *Pi*, nucleotide diversity; *S*, number of variable sites.

* Significant *p* value lower than .05 (*p* < .05).

** Significant *p* value lower than .01 (*p* < .01).

*** Significant *p* value lower than .001 (*p* < .001).

### 3.3 Genetic differentiation and structure

The AMOVA results indicated that the larger proportion of molecular genetic variation was found among populations (59.36%), and the remaining variation came within populations (40.64%). Exact tests showed significant variation on two levels (*p* < .001) (Table 5). The pairwise fixation (*F*_st_) and the gene flow (*N*_m_) are important index to evaluate the genetic differentiation of population (Hamrick et al., 1995; Kimura, 1979; Rousset, 1997). The value of *F*_st_ and *N*_m_ of seven different provinces based on paired sequences are estimated and listed in Table 6. The value of *F*_st_ and *N*_m_ ranged from 0 to 1.000, and 0 to 1.381, respectively. The results showed significant genetic differentiation between almost all pairs of populations except for four pairs, Yunnan and Shanghai, Yunnan and Jiangxi, Guangdong and Jiangxi, and Guangdong and Yunnan. The genetic differentiation between Sichuan and Shanghai, Hunan and Shanghai, and Hunan and Sichuan were highly significant. There was a frequent genetic flow between Shanghai and Yunnan pairs. In addition, the values of *N*_m_ showed a moderate genetic flow (*N*_m_ > 1) between three pairs (Jiangxi and Yunnan, Guangdong and Jiangxi, and Guangdong and Yunnan). The results showed there was a large genetic differentiation in the populations of these provinces, indicating, the gene flow among various populations is blocked (except for the regions mentioned above), resulting in obvious genetic differentiation (Whitlock & McCauley, 1999).

**TABLE 5:** Analysis of molecular variation (AMOVA) for the population of *Pomacea canaliculata* based on the concatenated genes.

Source of variation	df	Sum of squares	Variance components	Percentage of variation (%)	*p* Value
Among populations	6	232.556	6.25027 *V*_a_	59.36	<.001
Within populations	33	141.194	4.27862 *V*_b_	40.64	<.001
Total	39	373.750	10.52889		

*Note*: *V*_a_, *V*_b_: number of variance components.

**TABLE 6:** Comparison of the concatenated genes genetic differentiation pairwise genetic differentiation (*F*_st_: below diagonal) and gene flow (*N*_m_: above diagonal) among *Pomacea canaliculata* populations in seven regions in China.

Region	Shanghai	Jiangxi	Fujian	Yunnan	Sichuan	Guangdong	Hunan
Shanghai	–	0.369	0.197	**Inf.**	0	0.250	0
Jiangxi	**0.404**	–	0.288	**1.381**	0.031	**1.104**	0.139
Fujian	**0.559**	**0.465**	–	0.271	0.464	0.265	0.129
Yunnan	0	0.153	**0.480**	–	0.019	**2.083**	0.083
Sichuan	**1.000**	**0.889**	**0.350**	**0.931**	–	0.028	0
Guangdong	**0.500**	0.185	**0.486**	0.107	**0.900**	–	0.750
Hunan	**1.000**	**0.642**	**0.660**	**0.750**	**1.000**	**0.250**	–

*Note*: Inf.: shows that gene flow is full.

Bold values below diagonal represent high genetic differentiation, and bold values above diagonal represent high gene flow.

### 3.4 Population expansion analysis

**FIGURE 3:**
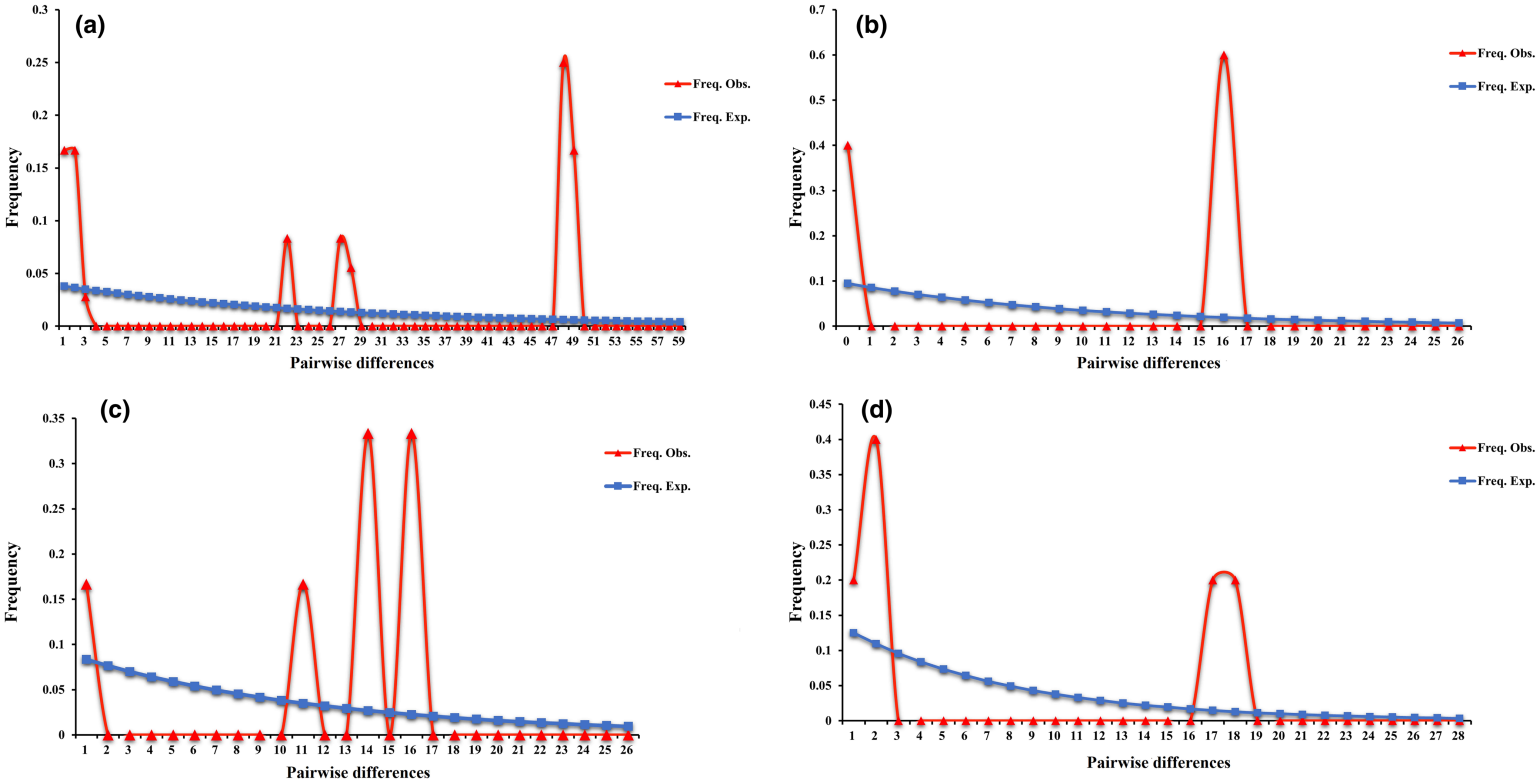
The mismatch distribution based on the concatenated genes for different populations of *Pomacea canaliculata*. (a) Population of Fujian; (b) population of Guangdong; (c) population of Jiangxi; (d) population of Yunnan.

The decline and growth of populations can leave genetic signatures detectable within individuals of a single population. Population decline can decrease sequence diversity, in contrast, population growth can favor conserved genes/sites (Harpending, 1994). Populations from Shanghai, Hunan and Sichuan lacked Tajima's *D* values in both single and concatenated genes. For single *cox1*, *nad1*, and the concatenated genes, the Tajima's *D* values of single and total populations (excluding Yunnan) were all positive, especially for Fujian and Guangdong populations, which showed a significant or highly significant values (*p* < .05 or *p* < .01), while the Tajima's *D* values of Yunnan population showed negative but insignificant values (Table 4). For single *nad4* gene, the values of Jiangxi and Yunnan populations were negative but insignificant, while other regions, Fujian and Guangdong, showed positive and significant values. Similarly, there were no Fu's *Fs* values in Shanghai, Sichuan, and Hunan. In contrast to the Tajima's *D* values in rest populations, the Fu's *Fs* values of total and single population on the basis of single gene or concatenated genes were all positive (Table 4). The values of Fujian population were significant in all single gene and concatenated genes, but the value of Guangdong population was only significant in *nad4* gene and concatenated genes. The results of neutrality tests in Guangdong and Fujian showed that these two regions might deviate from the neutral mutation. Mismatch distributions of Fujian, Guangdong, Jiangxi, and Yunnan (Figure 3) all presented the same pattern, multimodal, suggesting that *P. canaliculata* did not undergo demographic expansion in those regions.

### 3.5 Genetic distance analysis

The total genetic distances of haplotypes based on *cox1*, *nad1*, and *nad4* sequences of *P. canaliculata* were between 0.4% and 6.8% (Table 7). In contrast, the distances among *P. maculata*, *P. occulta*, and *P. diffusa* ranged from 6.5% to 27.2%. The genetic distance in *nad4* gene was the longest with the average of 3.7%, followed by *cox1* gene (3.4%), and the distance of *nad1* gene was the least, which is only 1.9%. However, the genetic distances of *P. maculata*, *P. occulta*, and *P. diffusa* based on *cox1*, *nad1*, and *nad4* genes were different from those in *P. canaliculata*, and the genetic distance based on *cox1* in interspecies was the smallest. Results suggested that *cox1* may be more conserved among interspecies of *Pomacea*, while *nad1* gene seemed to be more conserved in intraspecies of *P. canaliculata*. In addition, *nad4* gene was varied in both inter‐ and intraspecies.

**TABLE 7:** Analyze the K2P genetic distance between populations of *Pomacea canaliculata* and different species of *Pomacea* based on the haplotype of *cox1*, *nad1*, *nad4* genes.

Haplotype	A1	A2	A3	A4	A5	Hap_maculata	Hap_occulta
A1							
A2	0.009						
A3	0.047	0.057					
A4	0.011	0.016	0.054				
A5	0.042	0.052	0.004	0.049			
Hap_maculata	0.079	0.087	0.074	0.087	0.069		
Hap_occulta	0.059	0.069	0.069	0.066	0.064	0.072	
Hap_diffusa	0.182	0.182	0.185	0.191	0.185	0.185	0.170

Haplotype	B1	B2	B3	B4	B5	Hap_maculata	Hap_occulta
B1							
B2	0.006						
B3	0.009	0.015					
B4	0.021	0.027	0.024				
B5	0.024	0.030	0.027	0.003			
Hap_maculata	0.090	0.090	0.094	0.100	0.104		
Hap_occulta	0.090	0.090	0.094	0.097	0.101	0.056	
Hap_diffusa	0.190	0.190	0.190	0.206	0.210	0.186	0.190

Haplotype	C1	C2	C3	C4	C5	Hap_maculata	Hap_occulta
C1							
C2	0.023						
C3	0.023	0.015					
C4	0.072	0.059	0.063				
C5	0.027	0.019	0.004	0.068			
Hap_maculata	0.107	0.107	0.112	0.145	0.107		
Hap_occulta	0.135	0.140	0.140	0.160	0.135	0.081	
Hap_diffusa	0.272	0.272	0.266	0.289	0.260	0.238	0.232

### 3.6 Phylogenetic analysis

**FIGURE 4:**
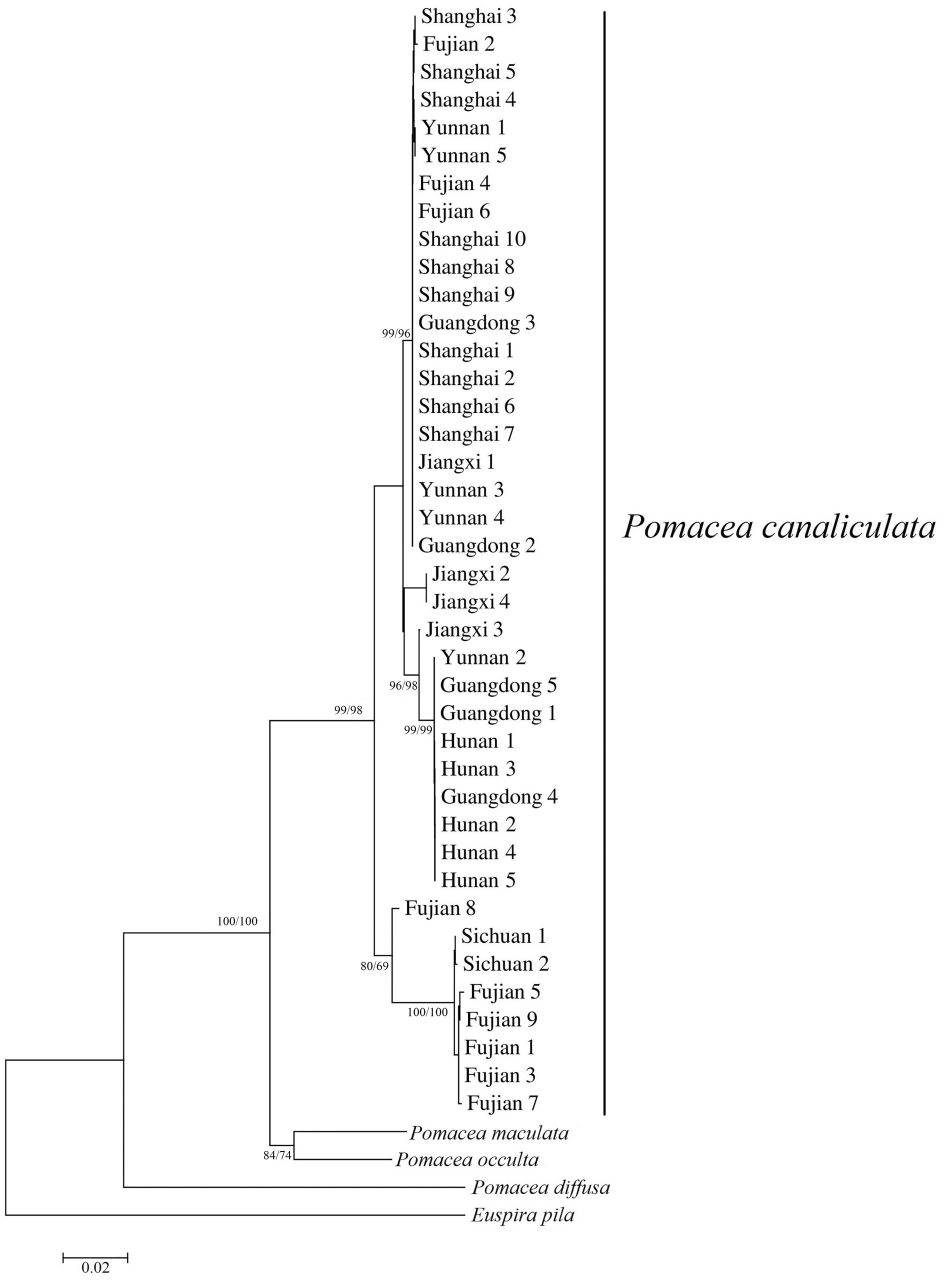
Phylogenetic relationships based on the concatenated genes.

In order to study the phylogenetic relationship of apple snails in seven regions of China, we constructed phylogenetic trees using the concatenated gene set for all populations. NJ and ML analyses produced phylogenetic trees with the same topology but with different bootstrap support values (Figure 4). All *P. canaliculata* samples grouped into one cluster, comprised by two obvious clades. One clade was grouped by samples from six different regions, including Shanghai, Fujian, Yunnan, Guangdong, Jiangxi, and Hunan. Another clade was formed by only two regions, Fujian and Sichuan. Results supported the monophyly of *P. canaliculata* with high statistic data (Bf = 100). Within *P. canaliculata*, individuals from Sichuan have a closer relationship with those from Fujian, and individuals collected from other six regions were more related. Interestingly, individuals from Fujian grouped into two different clusters. Compared to the distance within each cluster, the distance between FJ8 and other individuals was longer than others, suggesting there may be three evolutionary trends in golden apple snails in Fujian.

## 4 DISCUSSION

The present study used three mitochondrial genes to investigate the genetic diversity and differentiation, genetic distance, population expansion, and phylogenetic relationships of *P. canaliculata* collected from seven provinces in China. In 1979, apple snails were initially introduced to Taiwan as protein sources, and in 1981 they were introduced to Chinese mainland (Lv et al., 2013). They are considered an invasive alien species in China due to their strong fertility and high adaptivity (Mao et al., 2018). Usually, invasive alien species experience a bottleneck when invading new regions, and their genetic variation within the newly colonized areas is expected to decrease (Dlugosch & Parker, 2008).

The high proportion of shared haplotypes indicated that *P. canaliculata* has been an invasive species in China for a long time, and there may exist one ancestral haplotype within those regions based on both single gene and concatenated genes. For the possible ancestral haplotype, results suggested that Shanghai might constitute the ancestral invaded range of *P. canaliculata* because the ten individuals collected from Shanghai were all contained in the central haplotype. The present study showed that there were several different haplotypes distributed in four populations of *P. canaliculata* in seven regions, and the generation of some unique haplotypes may result from adaptation to the local environment (Dong et al., 2011). Additionally, the results found that individuals in Shanghai, Sichuan, and Hunan populations had only one haplotype, suggesting individuals in those regions may be introduced as single introductions, similar to the results obtained by Chuong et al. (2008) in Hawaii (Chuong et al., 2008). Of course, this phenomenon may also occur due to insufficient sampling in those regions. Low levels of haplotype diversity and nucleotide diversity are related to the founder effect, in which invasive species generate extreme genetic drift and bottleneck effects (Dlugosch & Parker, 2008). The populations in Yunnan and Guangdong also showed low haplotype diversity and nucleotide diversity, suggesting the possible existence of a founder effect in those provinces. However, the *P. canaliculata* populations in Fujian may not fit the above situation because high haplotype diversity and nucleotide differences are present in the Fujian population, and at least five samples exhibit unique haplotypes based on *cox1* and the concatenated genes. Based on neutrality tests and mismatch distribution analysis, the populations in all different regions, including Fujian, have reached a dynamic equilibrium without experiencing significant population expansion.

The *P. canaliculata* samples from different provinces formed two distinct clades in the phylogenetic tree, indicating golden apple snails have different geographical lineages in China, and all populations are single introductions except for the Fujian population. Nine individuals from Fujian were divided into two distinct clusters, and the distances between Fujian8 and other individuals are greater, indicating there may be multiple introductions of *P. canaliculata* in Fujian province, as previous studies suggested (Dlugosch & Parker, 2008; Guo & Zhang, 2014; Sekino et al., 2002). Our results support the conclusions of Hayes et al. (2008), and Yang, Liu, He, and Yu (2018) that multiple sources were involved in the initial introduction from South America (Hayes et al., 2008; Yang, Liu, He, & Yu, 2018). This scenario lead to increased genetic diversity among the introduced populations compared to a single source introduction, potentially facilitating establishment despite population bottleneck (Simon et al., 2011).

Following Wright's criterion for genetic differentiation, the value of *F*_st_ < 0.05 is defined as low genetic differentiation, 0.05 < *F*_st_ < 0.15 is moderate, *F*_st_ > 0.15 is high, and the value of *F*_st_ > 0.25 is very high genetic differentiation (Wright, 1984). Moreover, following the criterion of gene flow defined by Boivin et al. (2004), the rate of *N*_m_ < 1 is low genetic flow, values between 1 and 4 are high, and *N*_m_ > 4 is very high (Boivin et al., 2004). In the present study, most populations of *P. canaliculata* displayed significant genetic differentiation (*F*_st_ > 0.25) and low levels of gene flow (*N*_m_ < 1), with the majority of genetic diversification occurring between populations. Most sampling locations in this experiment are distantly located from each other. As a result, there is little opportunity for gene flow between populations, and significant genetic diversification has gradually developed by virtue of populations in different geographical regions being exposed to varying environmental conditions (Tian et al., 2018; Zheng et al., 2013). Or *F*_st_ is high due to the populations in China being introduced from different sources in South America (Hayes et al., 2008; Yang, Liu, He, & Yu, 2018). However, although there is large geographical distance between Yunnan and Shanghai, significant high gene flow was observed between those two populations, which contrasts with the hypothesis that gene flow is mainly associated with water flow (Lv et al., 2013). With well‐developed transportation networks between these two cities, human transport may be the dominant factor influencing gene flow as opposed water flow (Lv et al., 2013; Thaewnon‐Ngiw et al., 2003; Yang, Liu, He, & Yu, 2018).

According to Hebert, using *cox1* gene for species identification should base on the following criteria: (i) the intraspecific genetic distance should be less than 3%, and (ii) the interspecific genetic distance should be significantly higher than the intraspecific distance (typically over 10 times) (Hebert et al., 2003). However, in our study, the mean genetic distance within *P. canaliculata* based on *cox1* gene was higher than 3%, and the genetic distance among *P. maculata*, *P. occulta* and *P. diffusa* did not meet the 10 times of intraspecies distance. Our results showed that *P. canaliculata* had certain genetic differentiation within the species, but the genetic difference between *P. canaliculata* and other *Pomacea* species was small. These data support the suggestion of Yang et al. (2016) that this may be related to the close relatedness of these *Pomacea* species (Yang et al., 2016). The previous study suggested that *cox1*, *cytb*, and *cox3* are the most conserved mitochondrial genes within the genus *Pomacea* (Yang, Liu, Song, et al., 2018), similarly, the present study also indicated *cox1* gene is the most conserved gene among *Pomacea* species. Interestingly, the results suggested *nad1* gene is more conserved than *cox1* gene within *P. canaliculata* with lower genetic differentiation, which can be used as molecular marker to identify and distinguish apple snails. Although *nad4* gene has been used for molecular marker to identify tapeworms (Gong et al., 2021), our findings did not suggest to apply this gene for apple snail due to its high genetic distance.

## 5 CONCLUSIONS

The present study contributes to our understanding of the genetic variation, population expansion, and phylogenetic relationships of *P. canaliculata* in China by comparing and analyzing mt *cox1*, *nad1* and *nad4* genes. Analyses of haplotypes and phylogenetic relationship suggested there is multiple introductions of *P. canaliculata* in China from different sources. The values of Tajima's *D* and Fu's *Fs* and the results of mismatch distribution indicated that golden apple snail populations in China did not experience expansion recently. The results also provide a new genetic marker (*nad1*) for the identification and genetic study of *P. canaliculata*. Further research is needed to explore this potential molecular marker. In addition, these results support that *P. canaliculata* may have been introduced multiple times in China, which provides more clarity regarding the invasion history of this species and contributes to a deeper understanding of the population genetics and evolutionary biology of *P. canaliculata*. Future studies should include larger sample sizes from the sampled populations to validate the population genetic and phylogenetic patterns identified in this study and to examine the major factors influencing the invasion history of *P. canaliculata*.

## AUTHOR CONTRIBUTIONS

**Xi‐Long Yi:** Writing – original draft (equal). **Jing Liu:** Investigation (equal); resources (equal). **Mei‐Ling Cao:** Validation (equal). **Jun Xiong:** Visualization (equal). **Yuan‐Ping Deng:** Validation (equal). **Hui‐Mei Wang:** Validation (equal). **Ping‐Ping Ma:** Visualization (equal). **Guo‐Hua Liu:** Supervision (equal). **Hua Yang:** Supervision (equal).

## CONFLICT OF INTEREST STATEMENT

The authors declare that they have no competing interests.

### OPEN RESEARCH BADGES

This article has earned an Open Data badge for making publicly available the digitally‐shareable data necessary to reproduce the reported results. The data is available at [https://www.ncbi.nlm.nih.gov/nuccore].

## Data Availability

The *cox1* sequences of per individual of have been deposited in GenBank (accession numbers OQ983595–OQ983634) (https://www.ncbi.nlm.nih.gov/nuccore/OQ983595–https://www.ncbi.nlm.nih.gov/nuccore/OQ983634). The *nad1* sequences of per individual of have been deposited in GenBank (accession numbers OQ982487–OQ982526) (https://www.ncbi.nlm.nih.gov/nuccore/OQ982487–https://www.ncbi.nlm.nih.gov/nuccore/OQ982526). The *nad4* sequences of per individual of have been deposited in GenBank (accession numbers OQ982527–OQ982566) (https://www.ncbi.nlm.nih.gov/nuccore/OQ982527–https://www.ncbi.nlm.nih.gov/nuccore/OQ982566).
